# Effect of rice straw and swine manure biochar on N_2_O emission from paddy soil

**DOI:** 10.1038/s41598-020-67705-z

**Published:** 2020-07-02

**Authors:** Zhanbiao Yang, Yi Yu, Rujing Hu, Xiaoxun Xu, Junren Xian, Yuanxiang Yang, Lixia Liu, Zhang Cheng

**Affiliations:** 1 College of Environment, Sichuan Agricultural University, Chengdu, 611130 China; 2 College of Veterinary Medicine, Sichuan Agricultural University, Chengdu, 611130 Sichuan China

**Keywords:** Agroecology, Geochemistry, Climate-change mitigation

## Abstract

We analyzed the effects of rice straw biochar (RSBC) and swine manure biochar (SMBC) on N_2_O emission from paddy soil. The biochars were added to soil at the rates of 1% and 5% (w/w), and N_2_O emission, soil properties and soil enzyme activities were determined at the elongation, heading and maturation stages of rice growth. The N_2_O flux started within 2 h of adding the biochar, and decreased significantly thereafter during the three growth stages. The cumulative N_2_O emission was suppressed by 45.14–73.96% following biochar application, and 5% SMBC resulted in the lowest cumulative emission. In addition, biochar application significantly increased soil pH, soil organic carbon (SOC), NO_3_^−^ levels and urease activity, and decreased soil NH_4_^+^ and nitrate reductase activity. Regression analysis indicated that cumulative N_2_O emission was correlated positively to NH_4_^+^, and negatively to soil pH, SOC and NO_3_^−^. SEM further revealed that biochar application weakened the denitrification process, and the NH_4_^+^ level had the most significant impact on N_2_O emission. Taken together, RSBC and SMBC regulated the nitrogen cycle in paddy soil and mitigated N_2_O emission by increasing soil pH, decreasing nitrate reductase activity and NH_4_^+^ content.

## Introduction

Nitrous oxide (N_2_O) is a strong greenhouse gas (GHG) that persists in the atmosphere for 120 years, and accelerates the depletion of the stratospheric ozone layer^1^. The major source of the rising global N_2_O levels is the excessive use of nitrogen (N) fertilizers in agriculture^2^. In fact, the agricultural ecosystem contributes approximately 60% of the global anthropogenic N_2_O^3^. Rice is the staple food of nearly 50% of the world's population, and therefore the one of the major crops cultivated large-scale. Although less compared to that of upland soil, the annual N_2_O emission by rice paddy soil in China is still high at approximately 93 Gg^4^. Therefore, it is necessary to devise novel agricultural management strategies to mitigate the emission of N_2_O.

Biochar is a charcoal-like substance formed by controlled pyrolysis of agricultural waste^5^, and acts as an effective sponge for the organic and inorganic contaminants in soil and water due to its high pH, surface area, porosity and surface charge, as well as presence of various functional groups^6^. It has gained considerable attention in recent years for enhancing C levels, improving fertility, and controlling GHG emission^7,8^. Although there is clear evidence that biochar application is an effective soil amendment method in paddy fields^9–11^, its influence on soil N_2_O emission is still inconsistent. For example, Wang et al.^12^ reported a significant inhibitory effect of biochar on N_2_O emission from rice paddy field, especially in the early incubation stage, which was supported by several follow-up studies^7,13^. In contrast, Lin et al.^14^ found that wheat straw biochar increased N_2_O emission from acidic paddy soil. Furthermore, Angst et al.^15^ indicated that the cumulative emission of N_2_O was not significantly affected by biochar treatment. Therefore, there are several potential factors that influence N_2_O emission from paddy soil.

Soils N_2_O emission is closely related to the nitrogen cycle, which mainly comprises of nitrification and denitrification^16^. In addition, microbial processes like heterotrophic nitrification, couple denitrification, and reduction of dissimilated nitrate to ammonia also increase N_2_O emission^17^. While nitrification is the predominant N_2_O-generating process in aerobic soils, denitrification decreases with enhanced oxygen availability^18^. Therefore, heterotrophic denitrification is the primary source of N_2_O emission from flooded rice fields^19^, and is dominated by specific microorganisms^20,21^. Abiotic factors such as pH, organic carbon content, nitrogen availability and enzymatic activity modulate the soil microbiota, and therefore indirectly affect nitrogen cycling and N_2_O emission^22^. In this process, biochar acts as a redox catalyst and play a neglectable potential role in N_2_O emission. Usually, biochar is alkaline and contains considerable amounts of soluble base cations, which can increase soil pH when application to soil^23^. Increased soil pH, in turn, affect soil nitrate reductase activity and consequently decrease the N_2_O product ratios through weakening denitrification intensity under anaerobic conditions. However, the relationship of nitrification and denitrification on N_2_O emissions is not straightforward when available N levels changed in soil^24^. Cao et al.^25^ reported that application of biochar reduced the leaching of NO_3_^−^ and accumulated concentration of NO_3_^−^ in soil. More recently, Maucieri, et al.^13^ reported a decrease of NH_4_^+^ in biochar amended soil due to higher adsorption. Therefore, as the substrate, available N affected by biochar application is the major driver for N_2_O emission. Although biochar increases soil alkalization, its potential regulatory effect on the causal relationship between nitrogen cycle and N_2_O emission is still unclear.

Since N_2_O emission from paddy soil following biochar amendment varies considerably, we hypothesized that the biochar type and application rate affect N_2_O emission by regulating soil pH, SOC, NH_4_^+^ and NO_3_^−^. Therefore, we analyzed N_2_O emission from paddy soil after treating it with rice straw biochar (RSBC) and swine manure biochar (SMBC), and determined the effect of biochar type and application rate on the physiochemical characteristics of the soil. In addition, the causal relationship between soil properties and N_2_O emission after biochar application was also investigated, and the causal pathways were tested by structural equation model (SEM).

## Methods

### Biochar and soil preparation

Rice straw and swine manure were loaded into different porcelain crucibles (height—6 cm and internal diameter—5.5 cm) that were then covered with lids and placed in a muffle furnace (M110 Thermo Scientific, America). The temperature of the furnace was increased at 15 °C min^−1^ to 500 °C, and maintained at this temperature for 2 h. The final biochar was broken into < 1 cm long chips, stored in sealed bag and dried. The paddy soil samples were taken from depths of 0–20 cm from an agricultural field in Chengdu, Sichuan, China (30° 70′ 56.1″ N, 103° 86′ 05.4″ E) that cultivated wheat and rice alternately. The soil pH was 6.42, with SOC 17.44 mg g^−1^, ammoniacal nitrogen (NH_4_) 2.97 µg g^−1^, nitrate nitrogen (NO_3_) 11.2 µg g^−1^, and nitrite nitrogen (NO_2_) 0.21 mg kg^−1^.

### Biochar application and rice cultivation

The experiment was conducted in the greenhouse of Sichuan Agriculture University, China between May to September, 2018. Cylindrical plastic pots (diameter 380 mm; height 400 mm) were filled with 6 kg soil sample and 1% and 5% (w/w) RSBC and SMBC respectively, with 55% water holding capacity (four experimental groups, see Table 1), or only the soil (control). After 7 days of incubation, deionized water was poured into the plots and the water level was kept 2–3 cm above the soil. Rice seedlings were transplanted to the pots at the end of May 2018, with three seedlings planted per pot. The pots with different soil/biochar mixtures were arranged as per randomized complete block design with three replicates per treatment. Compound fertilizer (N:P:K = 15:15:15) was added at the seedling stage, and deionized water was added till 2–3 cm above the soil surface. Three soil samples were collected from each pot at the elongation (June 28, 2018), heading (August 2, 2018) and maturation stages (September 11, 2018) of rice growth and mixed. One part was stored at 4 °C immediately for testing enzyme and N_2_O emission, and the remaining was air dried for physicochemical analysis.

**Table 1:** Experimental treatments of this study.

Treatments	Description
CK	No biochar application, original soil
1% RSBC	1% mass of rice straw biochar mixed with 99% mass of original soil
5% RSBC	5% mass of rice straw biochar mixed with 95% mass of original soil
1% SMBC	1% mass of swine manure biochar mixed with 99% mass of original soil
5% SMBC	5% mass of swine manure biochar mixed with 95% mass of original soil

### Soil N_2_O sampling and analysis

Fifty grams fresh soil samples were put into 250 ml culture bottles in triplicate, and sealed with perforated silica gel plug. A three-way valve was used to expose the contents of the bottles to the outside air, and the headspace was sealed using hot melt adhesive. The soil samples were saturated with sterile ultrapure water to a depth of 3 cm, and incubated at 25 °C for 30 days. Five empty bottles were similarly set up to measure baseline N_2_O levels. The N_2_O in the headspace was sampled at 2 h, and 1, 3, 5, 7, 14 and 30 days using a gas sampling bag. After each sample collection, the lids were opened for half an hour to ensure thorough gas exchange between the atmosphere and the inside of the bottle. The concentration of N_2_O was measured using a Gas Chromatograph with an Electron Capture Detector (Agilent Technology 7890B, USA).

The N_2_O fluxes (μg kg^−1^ soil d^−1^) were calculated using Eq. (1):

1 $$F = (C - C_{0} ) \times M \times V \times \frac{273}{{22.4 \times (273 + 25)}} \times \frac{1}{m} \times \frac{1}{T}$$

where *F* is the N_2_O flux (μg kg^−1^ soil d^−1^), *C* is the concentration measured by the gas chromatograph (ng nl^−1^), *C*_*0*_ is the concentration measured in the blank bottle (ng nl^−1^), *M* is the molecular weight of N_2_O (g mol^−1^), *V* is the volume of gas in the culture flask (L), m is the dry soil weight (g), and *T* is the sampling interval (d).

The cumulative emission of soil N_2_O were calculated using Eq. (2):

2 $$E = \sum\limits_{{i - 1}}^{n} {\left( {\frac{{F_{{i - 1}} + F_{i} }}{2}} \right)} \times (t_{i} - t_{{i - 1}} )$$

where *E* is the cumulative emission of soil N_2_O (μg kg^−1^ soil d^−1^); *F* is the N_2_O fluxes (μg kg^−1^ soil d^−1^); *t*_*i*_ is the ith sampling time (d).

### Physicochemical analysis of biochar samples

The pH of the biochars was determined using a pH-meter (ST2100, OHAUS, America) and the solid to water ratio was set at 1:10 (1 g 10 ml^−1^). The ash contents of biochar were calculated by mass difference after burning in a muffle furnace at 600 °C for 8 h. Cation exchange capacity (CEC) was determined by the barium chloride (BaCl_2_) method. The content of carbon (C), nitrogen (N), hydrogen (H) and sulfur (S) were measured using an element analyzer (vario EL cube, ELEMENTAR, German). Surface area (S_BET_) and total pore volume (V_total_) was determined using a NOVA 1,200 surface area pore analyzer (Quantachrome Instruments, Boynton Beach, Florida, USA).

## Soil sample analysis

Soil pH was determined using a pH-meter (ST2100, OHAUS, America) at the solid-water ratio of 1:2.5 (5 g 12.5 ml^−1^). The concentration of NO_3_^−^, NO_2_^−^ and NH_4_^+^ were respectively determined by the phenol disulfonic acid method, sulfa/naphthalene ethylenediamine hydrochloride colorimetry and sodium phenol hypochlorite colorimetry using a UV spectrophotometer (UV-1800, MAPADA, China) respectively. SOC was determined by the potassium bichromate-ferrous sulfate titration method. Soil nitrate reductase (NR) activity was determined by phenol disulfonic acid method, and urease activity (UR) by the sodium phenate-sodium hypochlorite colorimetric method. All these methods have been described by Lu^26^.

### Statistical analysis

One-way analysis of variance (ANOVA) and Duncan test were used to compare the indices and treatments. Two-way ANOVA was used to test the effect of biochar type and rate on various indices. Regression analysis was used to explore the relationship between pH, SOC, NH_4_^+^, NO_3_^−^ and N_2_O emissions. Principal component analysis (PCA) and redundancy analysis (RDA) were performed to analyze the differences between biochar treatments, and the relationship between soil physico-chemistry and N_2_O emission. Multivariate analyses were performed using CANOCO version 5.0 for Windows. Exploratory path analysis was used to test the causal relationship between soil physico-chemistry, soil enzymes and N_2_O emission under different biochar types and application rates. SEM analyses were performed with IBM SPSS Amos 22.0 (IBM, New York, USA).

## Results

### Soil characteristics

**Figure 1:**
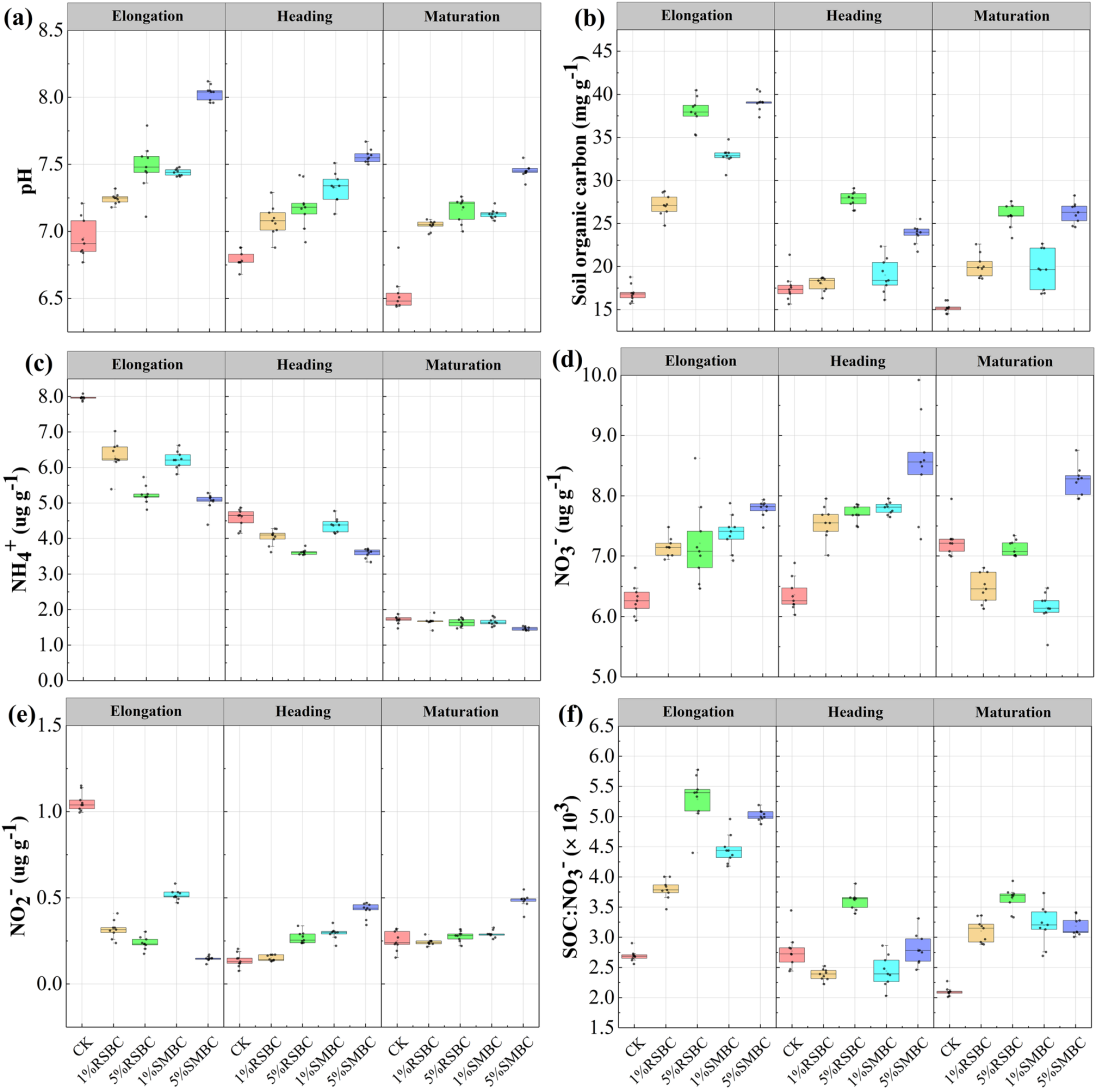
Soil physiochemical parameters during rice growth among treatments. (**a**), soil pH; (**b**), soil organic carbon; (**c**), soil NH_4_^+^ concentration; (**d**), soil NO_3_^−^ concentration; (**e**), soil NO_2_^−^ concentration; (**f**), rate of soil SOC: NO_3_^−^.

The physiochemical parameters of the paddy soil during the different rice growth stages are shown in Fig. 1. The pH value increased in the 1% RSBC, 1% SMBC, 5% RSBC and 5% SMBC-supplemented soils in that order compared to the control samples. Thus, SMBC had a greater alkalization effect than RSBC at both application rates (Fig. 1a). In addition, the soil pH during rice growth was highest with the addition of 5% SMBC due to its higher ash content and CEC (see Supplementary Table S1). The SOC content in the different groups ranged from 16.92–39.08, 17.59–23.80 and 15.23–26.26 mg g^−1^ during the elongation, heading and maturation stages respectively (Fig. 1b), and was higher in the biochar-supplemented soil compared to the control soil, indicating that biochar also retarded soil mineralization. Furthermore, addition of SMBC resulted in greater SOC compared to RSBC.

Soil NH_4_^+^, NO_3_^−^ and NO_2_^−^ levels were also significantly influenced by biochar application (see Supplementary Table S2, Fig. 1c,d). The NH_4_^+^ levels were significantly higher in the control soil samples relative to the biochar-treated soil during the elongation and heading stages, and the difference between the control and SMBC groups was always significant during the maturation stage (*P* < 0.05, see Supplementary Table S2). In contrast, biochar application significantly enhanced the NO_3_^−^ levels, and 5% SMBC resulted in maximum increase during all stages of growth. Consistently, the NO_3_^−^ levels were highest in the SMBC-treated compared to other treated soils during the maturation stage (Fig. 1d). The soil NO_2_^−^ levels ranged from 0.15 to 1.03, 0.13 to 0.43 and 0.25 to 0.49 µg g^−1^ respectively in the elongation, heading and maturation stages. In the elongation stage, 5% SMBC minimized NO_2_^−^ levels, which increased again during the heading and mature stages (Fig. 1e). Finally, biochar application significantly increased the SOC:NO_3_^−^ ratio compared to the control (*P* < 0.05, Fig. 1f) depending on the application rate. Taken together, biochar induces significant changes in the physicochemical characteristics of soil, which likely affect the rate of N_2_O emission.

### Soil enzymes activity

**Figure 2:**
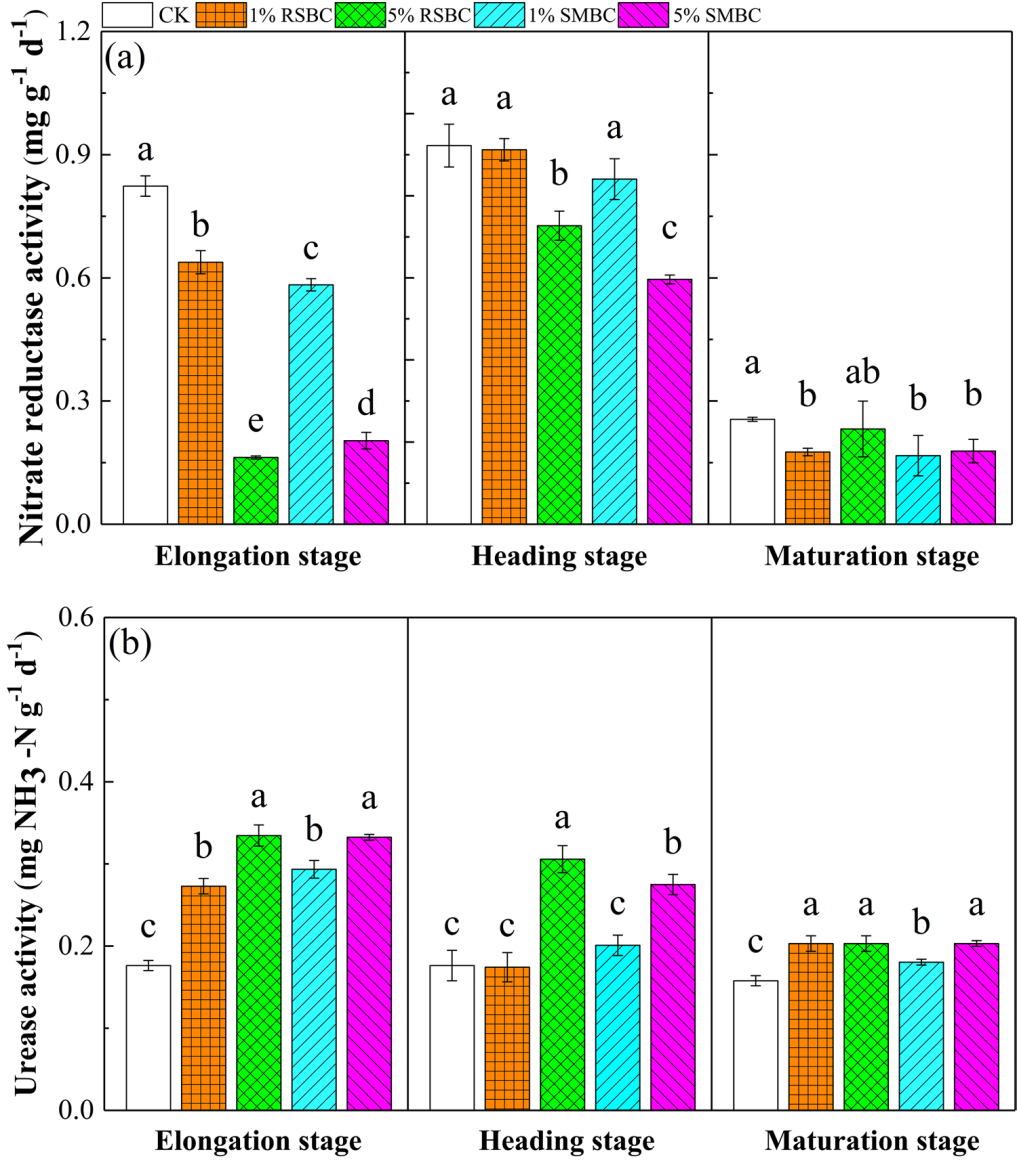
Effect of biochar application on soil nitrate reductase (**a**) and urease activity (**b**) in paddy soil at rice growth stages. Different letters above columns indicate significant differences at *P* < 0.05. Errorbar represented standard error of mean (n = 9).

The activities of nitrate reductase (NR) and urease (UR) differed between the control and biochar-supplemented soils, as well as between the RSBC and SMBC-treated samples. As shown in Fig. 2, biochar application markedly inhibited NR activity during the elongation and heading stages of rice growth (*P* < 0.05), while no significant effect was seen in the maturation stage regardless of the biochar type and application rate (see Supplementary Table S2). In contrast, biochar application increased the soil UR activity compared to control, and consistent with the trends in NR activity, the effect of application rate was significant only during the elongation and heading stages (*P* < 0.05) and not in the maturation stage (see Supplementary Table S2).

### Soil N_2_O emission

**Figure 3:**
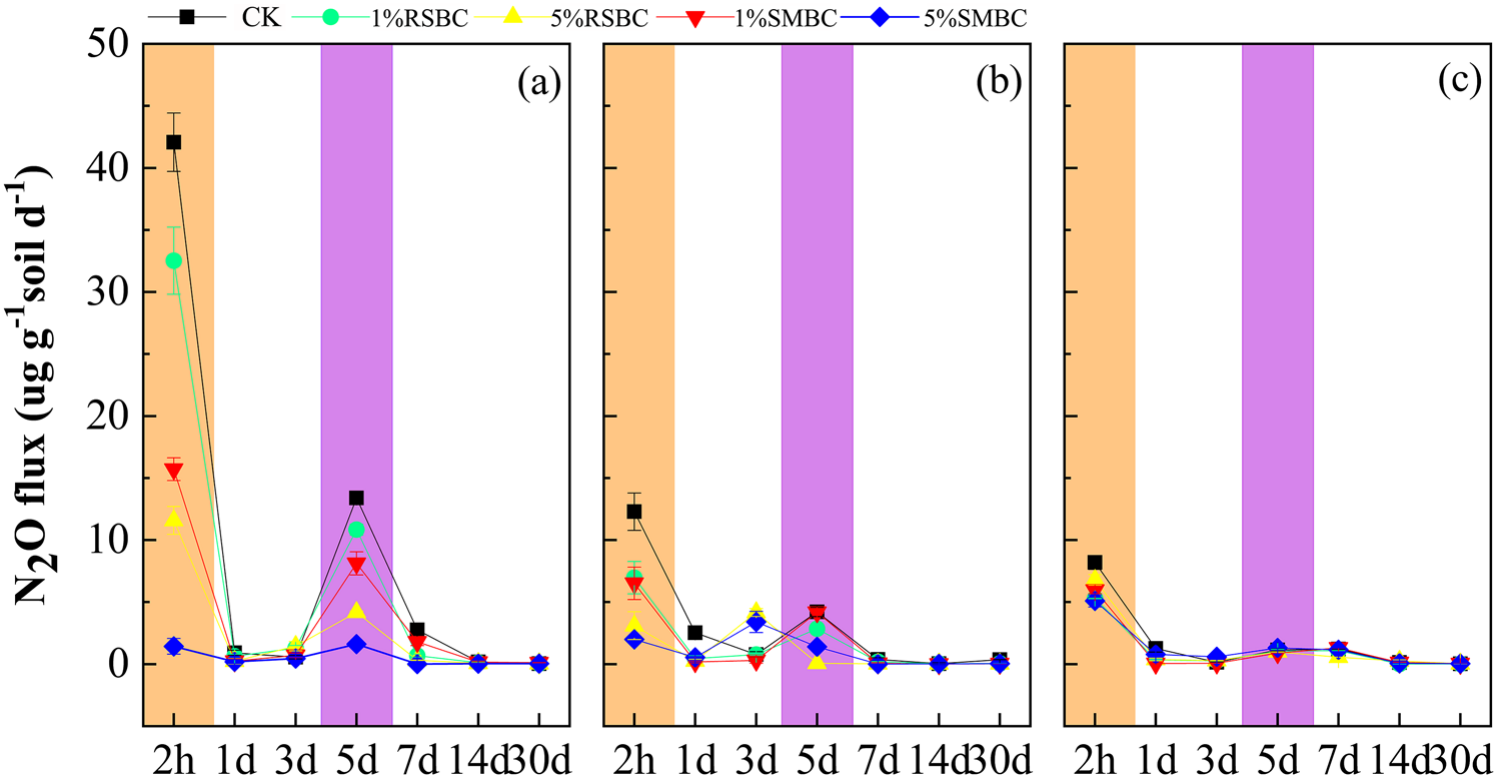
Soil N_2_O fluxes during 2 h-30 days of the incubation at elongation stage (**a**), heading stage (**b**), maturation stage (**c**). Errorbar represented standard error of mean (sem).

The N_2_O flux in the soil during the three growth stages of rice is shown in Fig. 3, which indicates a relatively consistent trend and a pulse-like pattern across all treatments. The N_2_O emission was highest 2 h after incubation and slowed after 5 days during all growth stages, indicating that the N_2_O flux primarily occurred soon after biochar application. At the elongation stage, N_2_O flux peaked at 2 h and 5 days after incubation. In addition, the highest N_2_O flux was seen in the control soils lacking biochar, and decreased in the 1% RSBC, 1% SMBC, 5% RSBC and 5% SMBC-supplemented soils in that order, which clearly indicated that adding more biochar lowered N_2_O emission. At the heading and maturation stages, the largest N_2_O flux was seen in the control samples 2 h after incubation. Interestingly, no significant differences were seen between the RSBC and SMBC groups after 1 day of incubation, suggesting that the effect of biochar on N_2_O emission is transient.

The cumulative N_2_O emission decreased significantly with biochar addition by 45.14–73.96% (*P* < 0.05; Table 2) compared to that of the control soil at all stages of growth. In addition, SMBC resulted in lower cumulative N_2_O emission compared to RSBC at the same application rate. The average cumulative N_2_O emission in the control, 1% RSBC, 1% SMBC, 5% RSBC and 5% SMBC samples were 123.1, 67.53, 64.63, 43.16 and 32.06 μg g^−1^ respectively. Thus, even after considering the difference between the various feedstocks, biochar derived from swine manure always showed better mitigation effect on N_2_O emission compared to that derived from rice straw.

**Table 2:** Cumulative emission of soil N_2_O during 2 h-30 days of the incubation at rice growth stage.

Treatment	Cumulative N_2_O emission (μg g^−1^ soil 30 d^−1^)			
	Elongation stage	Heading stage	Maturation stage	Cumulative N_2_O
CK	89.79 ± 0.51 a	19.34 ± 0.59 a	13.97 ± 0.27 a	123.10
1% RSBC	44.77 ± 1.78 b	12.53 ± 0.40 c	12.09 ± 0.21 c	67.53
5% RSBC	18.17 ± 0.45 d	10.68 ± 0.16 c	12.45 ± 0.08 c	43.16
1% SMBC	36.16 ± 2.27 c	15.67 ± 0.16 b	12.81 ± 0.24 bc	64.63
5% SMBC	5.87 ± 0.24 e	12.58 ± 1.23 c	13.61 ± 0.48 ab	32.06

Different lowercase letters within a column indicate significant differences at *P* < 0.05. Data was represented by mean ± standard error of mean (n = 9).

### Relationship between soil properties and N_2_O emission

**Figure 4:**
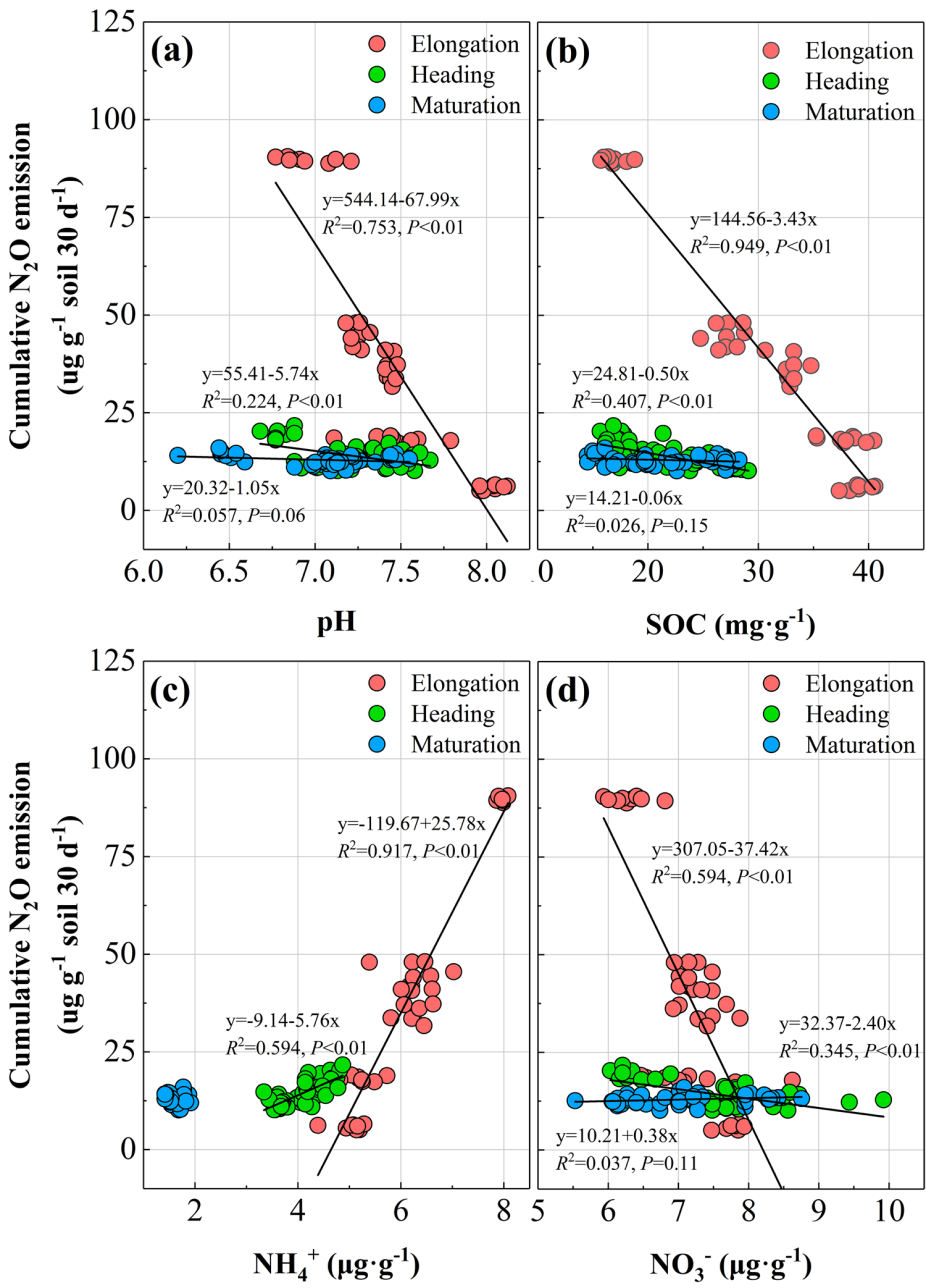
Regress analysis between soil properties and cumulative N_2_O emission during rice growth. (**a**), pH; (**b**), SOC; (**c**), NH_4_^+^; (**d**) NO_3_^−^.

Regression analysis showed that soil pH, SOC, NH_4_^+^ and NO_3_^−^ were significantly correlated to the cumulative N_2_O emission during the elongation and heading stages (Fig. 4). N_2_O emission was negatively correlated with pH, SOC and NO_3_^−^ levels, and positively correlated with soil NH_4_^+^ levels during elongation. The higher slope values in the regression equation demonstrated that N_2_O emission was highly sensitive to the soil indices, and peaked in the initial stages of rice growth before stabilizing in the heading and maturation stages.

**Figure 5:**
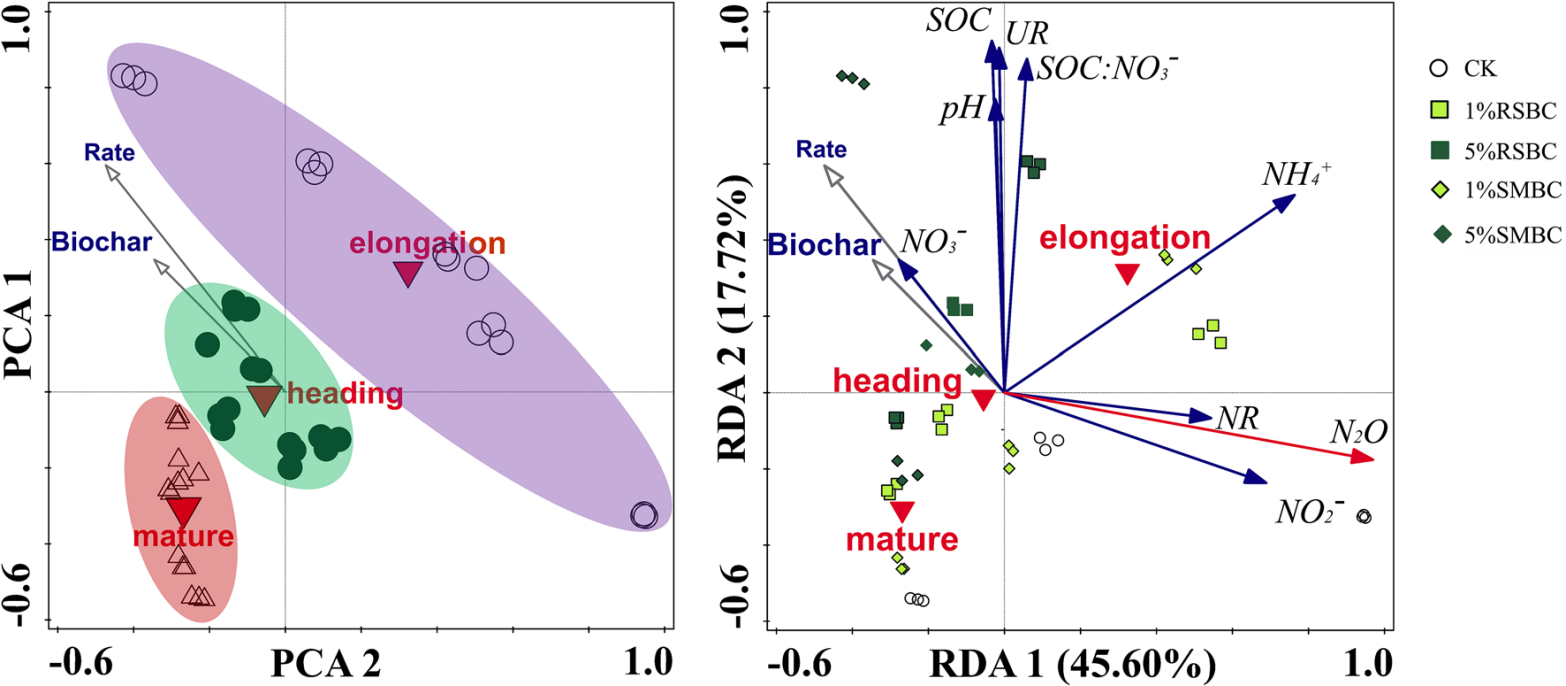
Principal Component Analysis (PCA) and redundancy Analysis (RDA) of the effect of biochar and application rate on cumulative N_2_O emission and soil physicochemical properties.

**Figure 6:**
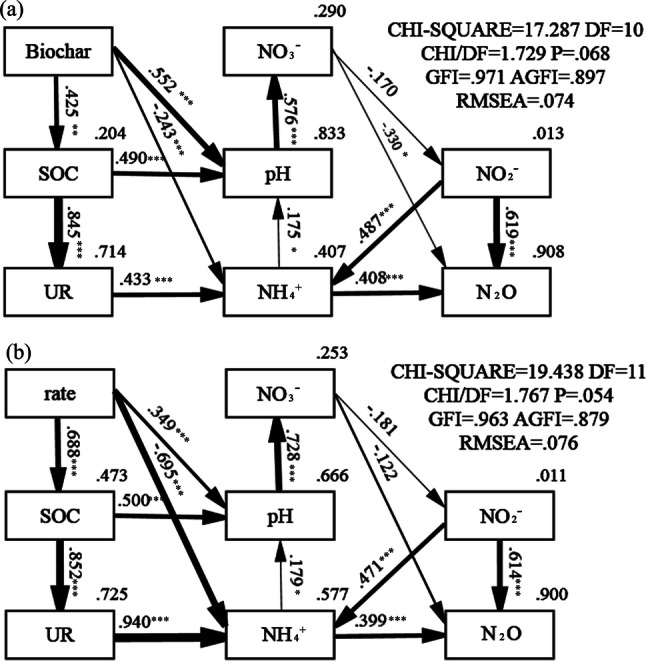
Structural equation model (SEM) diagram showing the potential causal pathways of biochar type (**a**) and application rate (**b**) on soil properties and N_2_O emission. The thickness of arrow represents the strength of the relationship between variables. The values associated with arrows are standardized pathway coefficients, and positive or negative numbers indicating the positive or negative relationships. Values in top right corner of box (endogenous variables) indicate the fraction be explained by the model.

**Figure 7:**
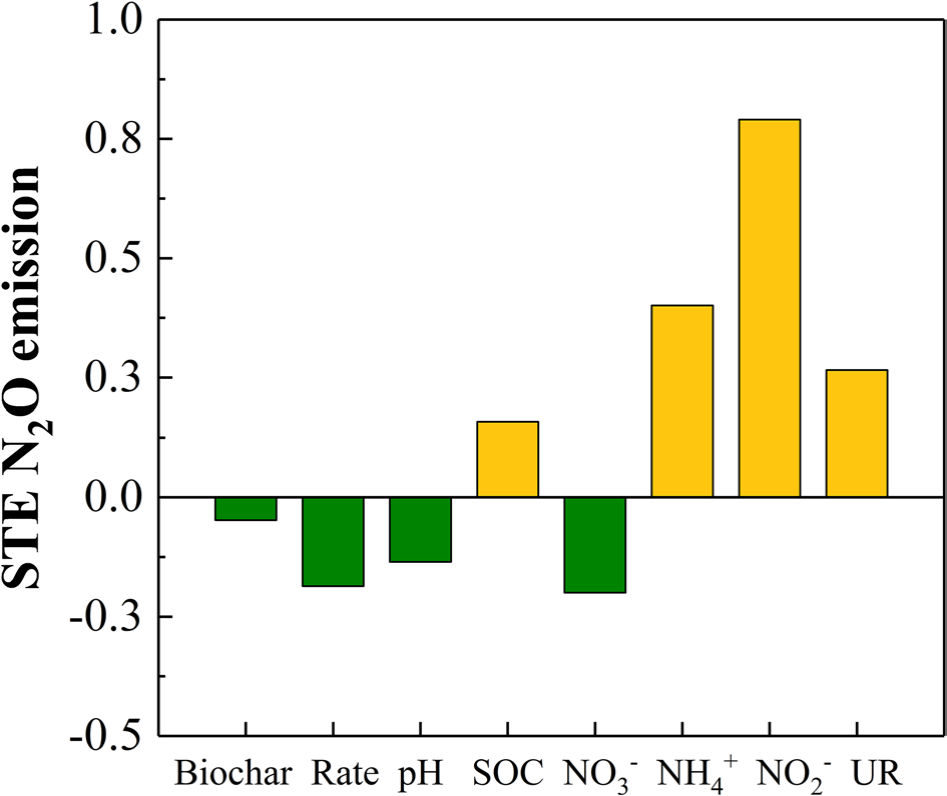
Standardized total effects (direct and indirect effects derived from the structural equation) of N_2_O emission.

The PCA analysis indicated that biochar application significantly affected N_2_O emission and soil properties, especially in the elongation stage (Fig. 5). RDA further showed that the first and second axes accounted for 45.6% and 17.72% of the total variation in the cumulative N_2_O emission (pseudo-F = 24.9, *P* = 0.002; Fig. 5). Biochar type and application rate significantly affected the cumulative N_2_O emission, which correlated positively with NO_2_^−^ and NH_4_^+^ levels and the NR activity, and negatively with NO_3_^−^, pH and SOC. Modified SEM was performed to evaluate the potential causal pathways of biochar type and application rate on N_2_O emission (Fig. 6). Fit statistics for the modified SEM showed an acceptable fit of the model (*P* = 0.068 and 0.054, respectively). The soil properties explained 90.8% and 90% variations in N_2_O emission with different biochar types and application rates respectively (Fig. 6). In addition, NO_2_^−^ and NH_4_^+^ levels were the most important factors controlling N_2_O emission affected by biochar, pH urease activity and NO_3_^−^ concentration. The biochar type and application rate also strongly affected SOC, urease activity and pH, which in turn controlled the soil NH_4_^+^ and NO_3_^−^ levels. In contrast, NO_3_^−^ level had a relatively weaker effect on N_2_O emission (Fig. 6). The standardized total effect, i.e. the sum of direct and indirect effects, on N_2_O emission was highest for NO_2_^−^, followed by NH_4_^+^. Biochar type and application rate had a negative standardized total effect (STE) on N_2_O emission, with higher application rates resulting in greater suppressive effect regardless of the biochar type (Fig. 7).

## Discussion

### Effects of biochar on soil characteristics

Biochar is produced through pyrolysis under limited oxygen condition. The characteristics are largely affected by feedstock, pyrolysis temperature and pyrolysis time. In this study, biochar application increased soil pH by 0.3–1.09 units at elongation stage, 0.29–0.78 units at the heading stage, and by 0.57–0.97 units at the maturation stage. The difference in biochar characteristics was due to different feedstock. Although lower pH value was found in SMBC, the pH in SMBC amended soil was significantly higher than that in RSBC due to its higher ash content and CEC. Alkalization of the soil not only decreased the acidic functional groups during pyrolysis^27^, but also altered the composition of the microbial community and regulated microbial N availability, thereby affecting soil N_2_O emission^21^. We found that cumulative N_2_O emission correlated negatively with soil pH, and 5% SMBC resulted in maximum alkalization and therefore lowest N_2_O emission. A higher soil pH is also known to suppress the activity of nitrate reductase (NR) that converts NO_3_^−^ to NO_2_^–^^28^. Indeed, biochar addition significantly decreased NR activity, especially at 5% application rates, and increased NO_3_^−^ levels and decreased NO_2_^−^ levels and N_2_O emission. Thus, biochar-induced pH increase is the possible mechanism of lower N_2_O emission.

Among the soil properties and edaphic factors influencing the N_2_O emission, NH_4_^+^ act as reaction substrate of nitrification and play a important role in controlling N_2_O emission^29^. Consistent with this, both RSBC and SMBC decreased the availability of NH_4_^+^, which correlated with lower cumulative N_2_O emission. Previous studies have also reported that biochar reduces NH_4_^+^ availability in the soil^13,30,31^. The credible explain was higher adsorb capacity to NH_4_^+^ by biochar due to its more adsorption sites and larger surface area^32,33^. The lower NH_4_^+^ concentration in the biochar-treated soils indicated lack of nitrification substrate, resulting in decreased N_2_O emission.

N_3_O^−^ contents is the major reaction substrate of denitrification, especially in paddy soils with low oxygen content and sufficient water content. During the process of denitrification, NO_3_^−^ is converted to NO_2_^−^ by nitrate reductase, and NO_2_^−^ is then converted to N_2_O by nitrite reductase^25^. The contents of NO_3_^−^ were increased in both RSBC and SMBC. This result seems to contribute to the process of denitrification and increase N_2_O emission. Furthermore, we found a decrease in nitrate reductase activity due to higher soil pH after RSBC and SMBC application, thus leading to accumulation of N_3_O^−^^34^.

### Effects of biochar on N_2_O emission

N_2_O emission from paddy soil was rapid in the initial phase of adding biochar, with a major peak at 2 h and a minor peak 5 days after incubation. These trends were likely due to ammonia oxidation and linked nitrifier denitrification or denitrification pathway. Our findings are consistent with that of Maucieri et al*.*^13^, who reported increased carbon and nitrogen availability for nitrification and denitrification in the initial stage of incubation. Gradual consumption of the available N slowed the N_2_O emission with time. Wang et al.^23^ also reported that high levels of NO_3_^−^ supported substrate for N_2_O production via denitrification in the initial anaerobic incubation after biochar application. One day later, sharp decrease in available NO_3_^−^ leading to decrease in N_2_O emission. In this study, we also observed a steady decline in the N_2_O flux after 1 day. The N_2_O emission decreased continuously with further consumption of reaction substrate at heading and maturation stages.

Furthermore, the suppressive effect on cumulative N_2_O emission increased with higher application rate, and was better with SMBC compared to RSBC. Cao et al.^25^ reported that 1–4% biochar application could effectively decrease soil N_2_O emission by 17.8–19.2%. The decrease of N_2_O emission from soil increased with increasing application rate. We found the cumulative N_2_O emission decreased by 45.14–73.96% compared to that of the control soil at all stages of growth. The least cumulative N_2_O emission was seen in soils supplemented with 5% SMBC. The inconsistent effects of biochar on N_2_O emission, in previous study, can be due to the fact that the biochar feedstock, inherent soil properties are major determinants of the nitrogen cycle^14,24^. In this study, the biochar in fact indirectly affects N_2_O emission by increasing the pH, and decreasing NH_4_^+^ levels and nitrate reductase activity.

The effects of biochar application on N_2_O emission depend on nitrification and denitrification processes^17^. Our findings further indicated that NO_2_^−^ and NH_4_^+^ had direct effects on N_2_O emission. This is not surprising since both are substrates of N_2_O during nitrification and denitrification. In addition, the high path coefficient from NH_4_^+^ to N_2_O indicated significant direct effects of RSBC and SMBC. However, the effect of NO_3_^−^ was clearly weakened by RSBC and SMBC as indicated by the weak relationship between NO_3_^−^ and N_2_O (standardized path coefficients: 0.170 and 0.181), which explains the increase in NO_3_^−^ levels after biochar treatment. Thus, biochar application suppressed denitrification of NO_3_^−^ to N_2_O, which increased the effect of NH_4_^+^ levels on N_2_O emission in paddy soil. Taken together, N_2_O emission is not only the result of high pH and biochar-induced decrease in NH_4_^+^ levels, but also related to changes in NO_3_^−^ levels during denitrification.

## Conclusion

Application of either RSBC or SMBC reduced N_2_O flux during the elongation, heading and maturation stages of rice crop in paddy soil, and suppressed cumulative N_2_O emission by 45.14–73.96%, with 5% SMBC resulting in the lowest cumulative N_2_O emission. Biochar application increased soil pH, SOC content and NO_3_^−^ levels, and decreased soil NH_4_^+^ levels and nitrate reductase activity. Lower NH_4_^+^ content in the soil strongly affected N_2_O emission, indicating that biochar mitigated N_2_O emission from paddy soil by increasing soil pH, decreasing nitrate reductase and NH_4_^+^ content.

## Supplementary information

Supplementary information.
