# Supplementary material for: Effect of rice straw and swine manure biochar on N2O emission from paddy soil
Source: Sci Rep. 2020 Jul 2;10:10843. doi: 10.1038/s41598-020-67705-z (PMC7331641; doi:10.1038/s41598-020-67705-z)
Supplement: Supplementary file 1 — Supplementary information. [file 41598_2020_67705_MOESM1_ESM.pdf]

Effect of rice straw and swine manure biochar on N_2O emission from paddy soil

Zhanbiao Yang[1a*], Yi Yu[1a], Rujing Hu[1], Xiaoxun Xu[1], Junren Xian[1], Yuanxiang Yang[1], Lixia Liu[2], Zhang Cheng[1*]

[1] College of Environment, Sichuan Agricultural University, Chengdu 611130, China.

[2] College of Veterinary Medicine, Sichuan Agricultural University, Chengdu, 611130 Sichuan, China.

[a] These authors contributed equally to this work.

[*]email: yzb195@126.com; chengzhang2001@foxmail.com

Table S1. The physicochemical properties of biochars

	Unit	RSBC	SMBC
pH	/	10.173 ± 0.16 a	9.976 ± 0.03 a
Ash	%	39.916 ± 1.52 b	59.927 ± 1.61 a
CEC	cmol kg^{-1}	128.287 ± 4.27 b	137.793 ± 3.57 a
C	%	32.893 ± 0.81 b	43.807 ± 1.28 a
N	%	0.940 ± 0.02 b	1.513 ± 0.07 a
H	%	0.705 ± 0.01b	1.305 ± 0.04 a
S	%	0.375 ± 0.02 a	0.289 ± 0.03 b
C/N	/	35.274 ± 0.51 a	28.553 ± 0.74 b
C/H	/	46.255 ± 0.94 b	32.934 ± 0.65 b
S_{BET}	m^2 g^{-1}	301.821	187.539
V_{total}	cm^3 g^{-1}	0.084	0.038

RSBC: rice straw biochar; SWBC: swine manure biochar; CEC: cation exchange capacity; S_{BET}: surface area; V_{total}: total pore volume. Different lowercase letters above the row indicate a significant difference between RSBC and SMBC at $p < 0.05$.

Table S2. Two-way *ANOVA* of biochars type and rate on soil properties and N_2O emission

Stage	Items	Biochar			Rate			Biochar × Rate		
		F	*p*		F	*p*		F	*p*	
Elongation stage										
	pH	100.841	*p* <0.001	***	124.939	*p* <0.001	***	22.866	*p* <0.001	***
	NH_4^+	2.537	*p* =0.119	ns	147.047	*p* <0.001	***	.246	*p* =0.622	ns
	NO_3^-	11.203	*p* =0.002	**	4.558	*p* =0.047	*	1.996	*p* =0.165	ns
	NO_2^-	16.137	*p* <0.001	***	271.991	*p* <0.001	***	113.940	*p* <0.001	***
	SOC	69.158	*p* <0.001	***	418.617	*p* <0.001	***	30.253	*p* <0.001	***
	SOC:NO_3^-	6.520	*p* =0.015	**	175.817	*p* <0.001	***	35.937	*p* <0.001	***
	UR	.716	*p* =0.402	ns	23.383	*p* <0.001	***	1.368	*p* =0.249	ns
	NR	2.279	*p* =0.139	ns	3010.410	*p* <0.001	***	30.335	*p* <0.001	***
Heading stage										
	pH	74.418	*p* <0.001	***	21.775	*p* <0.001	***	3.281	*p* =0.078	ns
	NH_4^+	6.317	*p* =0.016	**	93.797	*p* <0.001	***	8.854	*p* =0.005	**
	NO_3^-	14.724	*p* <0.001	***	10.355	*p* =0.003	**	4.561	*p* =0.039	*
	NO_2^-	166.996	*p* <0.001	***	115.136	*p* <0.001	***	0.500	*p* =0.484	ns
	SOC	10.639	*p* =0.002	**	256.449	*p* <0.001	***	30.137	*p* <0.001	***
	SOC:NO_3^-	24.156	*p* <0.001	***	111.374	*p* <0.001	***	32.289	*p* <0.001	***
	UR	0.401	*p* =0.530	ns	74.836	*p* <0.001	***	5.950	*p* =0.019	*
	NR	34.592	*p* <0.001	***	126.955	*p* <0.001	***	12.870	*p* =0.001	**
Mature stage										
	pH	34.267	*p* <0.001	***	47.641	*p* <0.001	***	10.226	*p* =0.003	**
	NH_4^+	7.269	*p* =0.010	*	10.362	*p* =0.003	**	4.723	*p* =0.036	*
	NO_3^-	23.378	*p* <0.001	***	283.035	*p* <0.001	***	78.103	*p* <0.001	***
	NO_2^-	106.843	*p* <0.001	***	86.636	*p* <0.001	***	42.897	*p* <0.001	***
	SOC	0.004	*p* =0.952	ns	160.970	*p* <0.001	***	0.603	*p* =0.442	ns
	SOC:NO_3^-	6.622	*p* =0.014	*	14.073	*p* =0.001	**	15.796	*p* <0.001	***
	UR	4.949	*p* =0.051	ns	1.978	*p* =0.178	ns	0.289	*p* =0.490	ns
	NR	1.407	*p* =0.141	ns	2.380	*p* =0.116	ns	0.659	p =0.280	ns

Note: $^{*}p$<0.05, $^{**}p$<0.01, $^{***}p$<0.001, ns not significant.
